# Investigating changes in mortality attributable to heat and cold in Stockholm, Sweden

**DOI:** 10.1007/s00484-018-1556-9

**Published:** 2018-05-11

**Authors:** Daniel Oudin Åström, Kristie L Ebi, Ana Maria Vicedo-Cabrera, Antonio Gasparrini

**Affiliations:** 1 Occupational and Environmental Medicine, Umeå University, 90187 Umeå, Sweden; 2 Center for Health and the Global Environment, University of Washington, Seattle, WA USA; 3 Department of Public Health, Environments and Society, London School of Hygiene & Tropical Medicine, London, UK

## Abstract

Projections of temperature-related mortality rely upon exposure-response relationships using recent data. Analyzing long historical data and trends may extend knowledge of past and present impacts that may provide additional insight and improve future scenarios. We collected daily mean temperatures and daily all-cause mortality for the period 1901–2013 for Stockholm County, Sweden, and calculated the total attributable fraction of mortality due to non-optimal temperatures and quantified the contribution of cold and heat. Total mortality attributable to non-optimal temperatures varied between periods and cold consistently had a larger impact on mortality than heat. Cold-related attributable fraction (AF) remained stable over time whereas heat-related AF decreased. AF on cold days remained stable over time, which may indicate that mortality during colder months may not decline as temperatures increase in the future. More research is needed to enhance estimates of burdens related to cold and heat in the future.

**Electronic supplementary material:**

The online version of this article (10.1007/s00484-018-1556-9) contains supplementary material, which is available to authorized users.

## Background

Over the course of the twentieth century, mean temperatures increased in Stockholm, Sweden (SMHI 2018). The observed trend is projected to continue over this century (Field 2012; Nikulin et al. 2011), making climate change a key challenge to be faced by health systems over course of this century.

Recently developed methods quantify attribution of mortality across the entire temperature distribution, defining non-optimal modest and extreme temperatures above and below the minimum mortality temperature (MMT) (Gasparrini and Leone 2014). Gasparrini et al. (2015) quantified mortality in 13 countries and found cold contributing to a larger attributable fraction (AF) than heat.

In light of the ongoing climate change, the numbers of studies aiming at quantifying future risks of temperature-related mortality, in particular due to heat, are growing (Huang et al. 2011; Hajat et al. 2014; Petkova et al. 2014). Recently, Gasparrini et al. (2017) projected temperature-related mortality risks at the end of the century in over 400 locations for several climate change scenarios; depending on geographical location, average excess mortality would increase under high emission scenarios. Projections of temperature-related mortality rely upon exposure-response relationships derived from recent observed data; however, using historical data may provide additional insight in these complex relationships.

The aim of the current study was to investigate if mortality attributable due to non-optimal temperatures changed across the twentieth century in Stockholm County, Sweden. By analyzing historical data and trends, we extend knowledge of past and present impacts of temperature on health, which may provide additional insight and improve future scenarios.

## Methods

The dataset and methods used in the current study are described in more detail elsewhere. In short, we collected daily mean temperatures and daily all-cause mortality for the period 1901–2013 for Stockholm County, Sweden (Åström et al. 2013). The relationship between the daily mean temperature and mortality was investigated using a Poisson regression allowing for overdispersion, adjusting for day of week, pandemics, and public Swedish holidays as well as adjusting for long-time trends using a smooth function (natural cubic spline) with eight degrees of freedom per year. We used a distributed lag nonlinear model considering lag times of up to 21 days (14 days were considered in a sensitivity analysis) for six periods (1901–1919, 1920–1939, 1940–1959, 1960–1979, 1980–1999, 2000–2013). For each period, we calculated the total AF of mortality due to non-optimal temperatures and quantified the contribution of cold and heat below and above MMT. In addition, we investigated mortality due to modest and extreme low and high temperatures. Modest cold and heat mortality AFs were calculated from the period-specific MMT to the 2.5th/97.5th percentile, whereas extreme cold and heat mortality AFs were calculated for temperatures below the 2.5th and above the 97.5th percentile of temperature distribution (Gasparrini et al. 2015).

To investigate any potential trend over time in the AF estimates, we performed a weighted least square linear regression with the AF estimates as the dependent variable and time as the independent variable using weights that were equal to the inverse of the standard deviation.

R version 3.2.3 and package DLNM was used (Gasparrini 2011).

## Results

Descriptive statistics of daily mortality and mean temperatures for the periods are presented in Supplementary Table 1. Total mortality attributable to non-optimal temperatures varied substantially between periods, ranging from 3.1% during 1960–1979 to 8.6% during 1920–1939. Cold consistently had a substantially larger impact on mortality than heat, by at least a factor of 2.5. Cold-related AF ranged between 2.8 and 7.9%, whereas heat-related AFs were for all but the first period below 1%. During all periods, modestly cold days contributed to the largest attributable fractions.

**Fig. 1:**
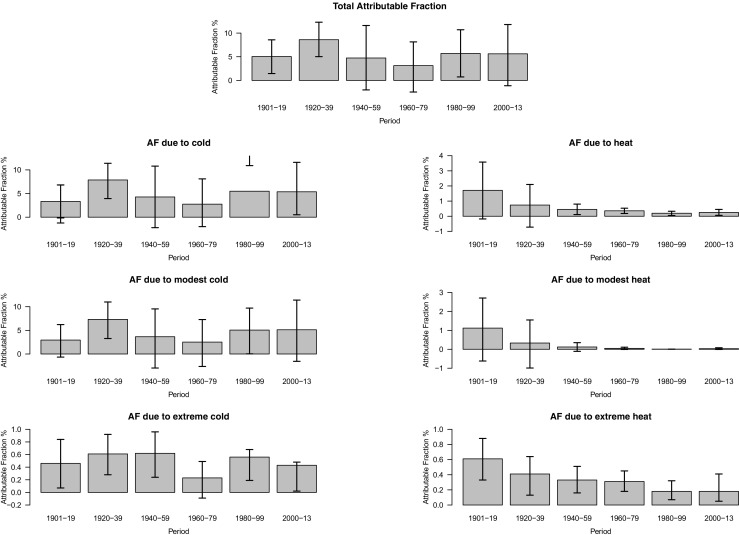
Attributable fractions due to non-optimal temperatures and estimates of trends over time presented with their 95% confidence intervals

There was no apparent trend over time for the cold-related AF, while there was some evidence suggesting a downward trend for heat-related AF. The decline over time in heat-related AF was driven by a decline in AF on modestly hot days, such that mortality during the latter periods was almost non-existent (Fig. 1, Supplementary Table 2). Supplementary Table 3 presents results using lag times of 14 days (results did not change).

## Discussion

For all periods, mortality attributable to cold was higher than that of heat. Mortality on modestly cold days accounted for the majority of mortality attributed to non-optimal temperature, which is in line with other studies (Gasparrini et al. 2015; Vicedo-Cabrera et al. 2018; Lee et al. 2018).

The estimates of the total AF for the last two periods are higher than those reported previously for Stockholm: Gasparrini et al. (2015) quantified a total AF of 3.9%, with cold accounting for 3.7% and heat for 0.2% in the Greater Stockholm area for the period 1990–2002 (our estimates for the same time period were total AF, 4.73%, with 4.51% due to cold and 0.22% due to heat). The present study used data from the county of Stockholm, which includes less urbanized settings and more disperse socioeconomic status on both an individual and neighborhood level.

We find modest evidence suggesting heat-related AF to be declining over time and strong evidence of a declining trend over time related to extreme heat. From the 1960s and onwards, the AF on modestly hot days was basically zero, which may partially be explained by increasing MMT and changes in the temperature-mortality relationships over time. Our findings of declining heat AF over time are in line with a recent study of 305 locations in ten countries, where the authors reported declining heat-related AFs over time in seven countries (Vicedo-Cabrera et al. 2018). Furthermore, Lee et al. (2018) reported decreasing heat-related AF over time in Taiwan and Korea, whereas it increased in Japan.

We find no strong evidence of a declining trend over time in cold-related AF. Cold-related AF peaked at 7.9% during the 1920s and 1930s, with more recent estimates of around 5.5% during the two latest periods of investigation. The magnitudes of the cold-related AFs, as well as the stable trend over time, show some similarities to those reported by Vicedo-Cabrera et al. (2018), implying that trends in cold-related impacts remain unclear.

It may not be appropriate to assume that higher temperatures would increase the number of heat-related deaths and decrease the number of cold-related deaths, nor may it be appropriate to not account for adaptation, which is likely to offset some of future increases in temperature-related mortality, as evidence suggests MMT not to be a stationary measure over time (Åström et al. 2016; Todd and Valleron 2015).

For Stockholm, Sweden, absolute and relative MMT increased over the course of the twentieth century (Åström et al. 2016), while the effect of extreme temperatures on mortality decreased (Åström et al. 2013), suggesting partial adaptation to increasing temperatures. Decreasing effects of temperature on mortality over long-time periods have been reported elsewhere (Davis et al. 2003a, b, Sheridan et al. 2009, Carson et al. 2006; Ekamper et al. 2009). However, it remains inevitably difficult to separate adaptation and acclimatization to increasing temperatures from general societal improvements associated with development, such as standard of living and the quality and accessibility of health care facilities (Sellers and Ebi 2017).

Using historic data, we found that the AF on cold days remained stable over time, which suggests that mortality during the colder months may not simply decline as temperatures increase in the future. How winter mortality will continue to evolve will remain a difficult component in the projections of future temperature-related mortality (Ebi and Mills 2013), as evidence suggests winter mortality may modify heat-related mortality the following summer (Rocklöv et al. 2009; Stafoggia et al. 2009).

With ongoing climate warming and associated adaptive processes, the temperature-mortality relationships on both sides of MMT may change, with the magnitude and direction of change uncertain. More research is needed to enhance estimates of the burden related to cold and heat in the future.

## Conclusion

There was substantial variation between periods over the course of 110 years in the percentage of deaths attributable to non-optimal temperatures in Stockholm County, Sweden. The majority of these deaths occurred during modestly cold days. The attributable fraction remained similar over time for cold and declined for heat.

## Electronic supplementary material

ESM 1 (DOCX 21 kb)
