# Supplementary material for: Investigating changes in mortality attributable to heat and cold in Stockholm, Sweden
Source: Int J Biometeorol. 2018 May 11;62(9):1777–80. doi: 10.1007/s00484-018-1556-9 (PMC6132879; doi:10.1007/s00484-018-1556-9)
Supplement: Supplementary file 1 — (DOCX 21 kb) [file 484_2018_1556_MOESM1_ESM.docx]

Supplementary Table 1. Descriptive statistics of daily morality and daily mean temperature for the different periods

|  | Daily Counts of Mortality | | | Daily Mean Temperature (°C) | | | | | MMT | |
| --- | --- | --- | --- | --- | --- | --- | --- | --- | --- | --- |
| Period | Mean | Min | Max | Min | 2.5^th*^ | Mean | 97.5^th*^ | Max | MMT_1_ | MMT_2_ |
| 1901-19 | 20.9 | 3 | 82 | -19.6 | -8.8 | 5.9 | 19.8 | 25.9 | 8.3 | 8.3 |
| 1920-39 | 22.7 | 5 | 53 | -16.4 | -7.9 | 6.5 | 20.2 | 26.4 | 11.2 | 11.3 |
| 1940-59 | 27.5 | 10 | 54 | -23.7 | -10.0 | 6.5 | 20.6 | 26.8 | 16.2 | 16.8 |
| 1960-79 | 37.2 | 14 | 72 | -21.2 | -9.1 | 6.6 | 20.6 | 28.3 | 18.0 | 15.9 |
| 1980-99 | 41.6 | 20 | 81 | -23.9 | -8.4 | 7.0 | 21.1 | 27.6 | 19.8 | 17.0 |
| 2000-13 | 42.4 | 20 | 75 | -17.1 | -7.5 | 7.9 | 21.6 | 26.7 | 18.8 | 14.6 |
| *: Percentiles of the period-specific temperature distribution MMT_1_: Minimum Mortality Temperature for the models using a lag structure of up to 21 days  MMT_2_: Minimum Mortality Temperature for the models using a lag structure of up to 14 days | | | | | | | | | | |

Supplementary Table 2. Attributable fractions due to non-optimal temperatures and estimates of trends over time presented with their 95% Confidence Intervals using a lag structure of up to 21 days.

| Period | Total | Cold | Heat | Modest Cold | Extreme Cold | Modest Heat | Extreme Heat |
| --- | --- | --- | --- | --- | --- | --- | --- |
| 1901-19 | 5.04 (1.44;8.57) | 3.33 (-0.16;6.81) | 1.71 (-0.18;3.58) | 2.95 (-0.66;6.22) | 0.46 (0.07;0.84) | 1.12 (-0.62;2.71) | 0.62 (0.33;0.88) |
| 1920-39 | 8.60 (5.02;12.2) | 7.87 (3.93;11.4) | 0.74 (-0.72;2.10) | 7.32 (3.27;11.0) | 0.61 (0.28;0.92) | 0.33 (-0.99;1.55) | 0.41 (0.13;0.64) |
| 1940-59 | 4.73 (-2.02;11.6) | 4.28 (-2.22;10.8) | 0.45 (0.11;0.80) | 3.65 (-2.96;9.54) | 0.62 (0.24;0.96) | 0.12 (-0.11;0.35) | 0.33 (0.16;0.51) |
| 1960-79 | 3.12 (-2.46;8.13) | 2.76 (-2.78;8.10) | 0.36 (0.18;0.53) | 2.52 (-2.63;7.28) | 0.23 (-0.09;0.49) | 0.05 (-0.01;0.11) | 0.31 (0.18;0.45) |
| 1980-99 | 5.69 (0.73;10.7) | 5.49 (0.51;10.9) | 0.20 (0.05;0.33) | 5.06 (0.03;9.71) | 0.45 (0.19;0.68) | 0.01 (-0.00;0.01) | 0.19 (0.07;0.32) |
| 2000-13 | 5.62 (-1.13;11.8) | 5.37 (-1.23;11.6) | 0.25 (0.06;0.45) | 5.12 (-1.55;11.4) | 0.26 (0.02;0.48) | 0.03 (-0.01;0.08) | 0.22 (0.05;0.41) |
| β_trend_ | -0.25 (-1.60;1.10) | 0.04 (-1.31;1.40) | -0.16 (-0.34;0.03) | 0.08 (-1.31;1.46) | -0.02 (-0.11;0.07) | -0.10 (-0.25;0.05) | -0.08 (-0.12;-0.04) |

Supplementary Table 3. Attributable fractions due to non-optimal temperatures and estimates of trends over time presented with their 95% Confidence Intervals using a lag structure of up to 14 days.

| Period | Total | Cold | Heat | Modest Cold | Extreme Cold | Modest Heat | Extreme Heat |
| --- | --- | --- | --- | --- | --- | --- | --- |
| 1901-19 | 4.59 (1.17;7.90) | 2.52 (-1.25;5.89) | 2.08 (1.01;3.05) | 2.08 (-1.19;5.66) | 0.45 (0.15;0.73) | 1.48 (0.62;2.37) | 0.64 (0.44;0.81) |
| 1920-39 | 5.00 (2.12;7.76) | 3.58 (0.17;6.75) | 1.42 (0.43;2.45) | 3.20 (-0.04;6.47) | 0.38 (0.12;0.62) | 0.83 (-0.11;1.76) | 0.61 (0.42;0.80) |
| 1940-59 | 5.01 (0.03;10.3) | 4.60 (-0.55;9.23) | 0.41 (0.18;0.60) | 4.01 (-1.16;9.05) | 0.58 (0.32;0.85) | 0.07 (-0.05;0.19) | 0.34 (0.21;0.47) |
| 1960-79 | 2.30 (-1.38;5.89) | 1.83 (-1.59;5.55) | 0.47 (0.20;0.74) | 1.68 (-2.14;5.29) | 0.14 (-0.08;0.38) | 0.13 (-0.05;0.30) | 0.35 (0.21;0.46) |
| 1980-99 | 3.36 (-0.04;6.62) | 3.27 (-0.15;6.58) | 0.09 (-0.11;0.28) | 3.01 (-0.19;6.07) | 0.27 (0.05;0.48) | -0.04 (-0.13;0.07) | 0.13 (0.00;0.24) |
| 2000-13 | 3.07 (-0.74;6.58) | 2.60 (-1.15;5.86) | 0.47 (-0.20;1.14) | 2.34 (-1.18;5.62) | 0.28 (0.03;0.50) | 0.24 (-0.33;0.81) | 0.24 (0.10;0.38) |
| β_trend_ | -0.43 (-0.98;0.11) | -0.08 (-0.72;0.56) | -0.29 (-0.64;0.06) | -0.04 (-0.60;0.53) | -0.04 (-0.14;0.05) | -0.17 (-0.47;0.12) | -0.10 (-0.17;-0.03) |
